# Intraoperative superior hypogastric plexus block for pain relief after a cesarean section: a case-control study

**DOI:** 10.3325/cmj.2021.62.472

**Published:** 2021-10

**Authors:** Hakan Peker, Kemal Atasayan, Berna Haliloglu Peker, Cetin Kilicci

**Affiliations:** 1 Nisantasi University Health Vocational School, Istanbul, Turkey; 2 Department of Obstetrics and Gynecology Bezmialem Vakıf University Faculty of Medicine, Istanbul, Turkey; 3 Department of Obstetrics and Gynecology Maltepe University Faculty of Medicine, Istanbul, Turkey; 4 Department of Obstetrics and Gynecology Zeynep Kamil Women and Children Diseases Education and Research Hospital, Istanbul, Turkey

## Abstract

**Aim:**

To investigate the efficacy of intraoperative superior hypogastric plexus (SHP) block for postoperative pain relief in patients undergoing a cesarean section.

**Methods:**

One hundred and fifteen pregnant women scheduled for an elective cesarean under general anesthesia were randomly divided into an SHP block (n = 65) and a control group (n = 50). SHP block was administered with bupivacaine injection. The controls received saline injection in the SHP area. Postoperative pain was assessed by the 10-cm visual analog scale (VAS). The presence of side effects and complications, including opioid or non-steroidal anti-inflammatory drugs (NSAID) requirement, gastrointestinal function, nausea, and vomiting were evaluated.

**Results:**

The SHP block group had significantly lower VAS scores 2, 6, 24, and 48 hours postoperatively (*P* < 0.001) and required a significantly lower rescue dose of NSAID or opioids (*P* = 0.003, *P* < 0.05, respectively).

**Conclusions:**

SHP block may be an effective and safe pain relief treatment after a cesarean section.

Cesarean section is a rescue surgical procedure mostly performed in order to save the fetus's life or avoid labor complications (1). Rising cesarean section rates worldwide, especially in middle-income and developed countries, are becoming a major public health problem (2,3).

Insufficient pain relief is a common complaint among inpatients. In a Spanish study, 30%-70% of the patients reported moderate to severe pain after surgery (4). Among women undergoing a cesarean section, at least 10.9% experience severe pain within 24 hours postoperatively (5). Cesarean section ranked ninth among 179 different surgical procedures according to pain severity on the first postoperative day (6).

In this regard, a combined postoperative pain management has been recommended for cesarean delivery. Effective analgesia is achieved by opioids administration either by patient-controlled analgesia or neuraxial injection (7-9). Different techniques have various advantages and disadvantages (10).

Superior hypogastric plexus (SHP) block has been used for treatment of postoperative pain after gynecologic procedures (11-13). The SHP is a retroperitoneal structure located bilaterally between the fifth lumbar and the first sacral vertebral body, which is easily reachable when performing blocks for relieving pelvic pain (14). The block can be performed under different guidance techniques, such as fluoroscopy-guidance, ultrasound-guidance, or computerized tomography-guidance (15). Furthermore, it can be easily carried out intraoperatively during abdominal surgery (11-13). In the light of this, we hypothesized that SHP block may be an alternative analgesic method to be used after a cesarean section. In the present study, we assessed the efficacy of intraoperative SHP block for pain relief after a cesarean section.

## Patients and methods

This case-control study was conducted at Zeynep Kamil Women and Children Diseases Education and Research Hospital between January and March 2019. The Institutional Review Board (101/5.5.2017) approved the study. Informed verbal and written consent were obtained from all participants.

### Patient selection

The study enrolled women aged between 18 and 40 years with term singleton pregnancies and no history of cesarean section or abdominal surgery scheduled for elective cesarean section. General anesthesia was performed to allow us to more accurately assess the procedure's efficacy in the early postoperative period. The study enrolled only Class 1-2 patients according to the American Society of Anesthesiologists (ASA) Classification. Patients were randomly allocated into two groups. The allocation sequence was generated by a random number table, and group allocation was concealed in sealed, opaque envelopes, which were not opened until operation. During the operation, the envelope was opened by a nurse outside the operating theater. The nurse prepared a blind syringe with the study drug, which was then transferred to a sterile bowl in the operating room and injected. Bupivacaine and saline are both colorless and not possible to identify by visual appearance or smell. The envelope was then sealed again and not opened until the study was concluded. The patients, anesthesiologists, and nurses providing postoperative care were blinded to group assignment. The control group received a saline injection in the SHP area. We excluded patients with bleeding disturbances, chronic pelvic pain, a history of opioid or NSAID allergy, asthma, diabetes, liver or kidney diseases, drug or alcohol abuse, and pregnancy-induced hypertension. The patients who underwent an emergency cesarean section were also excluded. The indications for primary elective cesarean were breech presentation and maternal fear of vaginal birth.

### Anesthesia

The routine standardized general anesthesia and perioperative analgesia protocol were applied without premedication. General anesthesia was induced by propofol 2.5 mg/kg and rocuronium 0.6 mg/kg. Following adequate muscle relaxation, endotracheal intubation was performed. Anesthesia was maintained by sevoflurane 0.6 MAC in oxygen/air. Sevoflurane concentration was adjusted according to the hemodynamic response. For perioperative analgesia, 15 mg/kg paracetamol (max 1 gr) and 20 mg tenoxicam were given intravenously approximately 30 minutes before the surgery ended.

### Surgery and the superior hypogastric block technique

**Figure 1:**
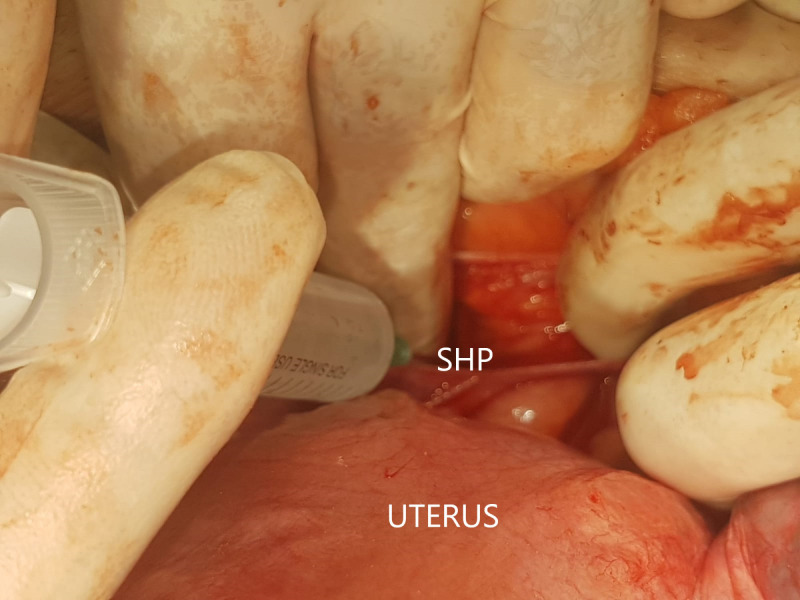
The technique of superior hypogastric plexus (SHP) block.

The surgery was carried out through a Pfannenstiel incision. After the newborn and placenta delivery, a uterine incision was closed by exteriorizing the uterus. Upon achieving hemostasis, the SHP block was carried out retroperitoneally following the injection of either 20 mL of bupivacaine 2.5 mg/mL or saline 9 mg/mL in the SHP area (11) (Figure 1).

At the post-anesthesia care unit (PACU), the women were monitored for the degree of sedation, hemodynamic and respiratory stability, pain, and nausea. When the patient was awake with stable vital signs, she was discharged to the ward. Postoperative pain was evaluated with the 10-cm visual analog scale (VAS), where 0 indicates no pain and 10 indicates the worst pain. The patients were asked to rate their pain when lying still in bed (at rest) and on movement. VAS scores were assessed and recorded by a pain nurse at 2, 6, 24, and 48 hours after surgery. The routinely used analgesics for pain relief in the PACU and surgical ward were the nonsteroid anti-inflammatory drug (NSAID) diclofenac sodium and the opioid pethidine. The VAS score ≥4 is considered a pain level that should be treated (16). The patients with VAS scores ≥4 were first administered diclofenac sodium. If there was no decrease in VAS scores or NSAID was inadequate, the opioid analgesic was administered. The number of vials of analgesics (diclofenac sodium 75 mg = 1 vial; pethidine HCl 50 mg = 1 vial) used was recorded. Nausea and vomiting were considered present when recorded at least once for 48 hours of the study. The time from surgery to the first passing of gas was recorded as a sign of bowel movements and the return of gastrointestinal function. The length of surgery was also noted.

The primary study outcome was the pain score at rest and on movement 24 hours after surgery. The secondary outcomes were the pain scores at rest and on movement 2, 6, and 48 hours after surgery; opioid or NSAID requirement; return of gastrointestinal function; the rate of nausea and vomiting as a side effect; and surgery duration.

A preliminary power analysis indicated that a sample size of 70 patients (35 for case group and 35 for control group) provided a statistical power (1-β) of at least 80% at α = 0.05 for the detection of 1-cm differences in VAS scores between the two groups at 24 hours after surgery.

### Statistical method

The normality of distribution was assessed with the Kolmogorov-Smirnov test. The parametric data were presented as means and standard deviations, the non-parametric data as median and range, ratio, and the categorical data as counts and percentages. The Mann-Whitney U test was used to compare the continuous data, while the χ^2^ test was used to compare the categorical data. The analysis was performed with SPSS for Windows, version 22.0 (IBM Corp., Armonk, NY, USA).

## RESULTS

**Figure 2:**
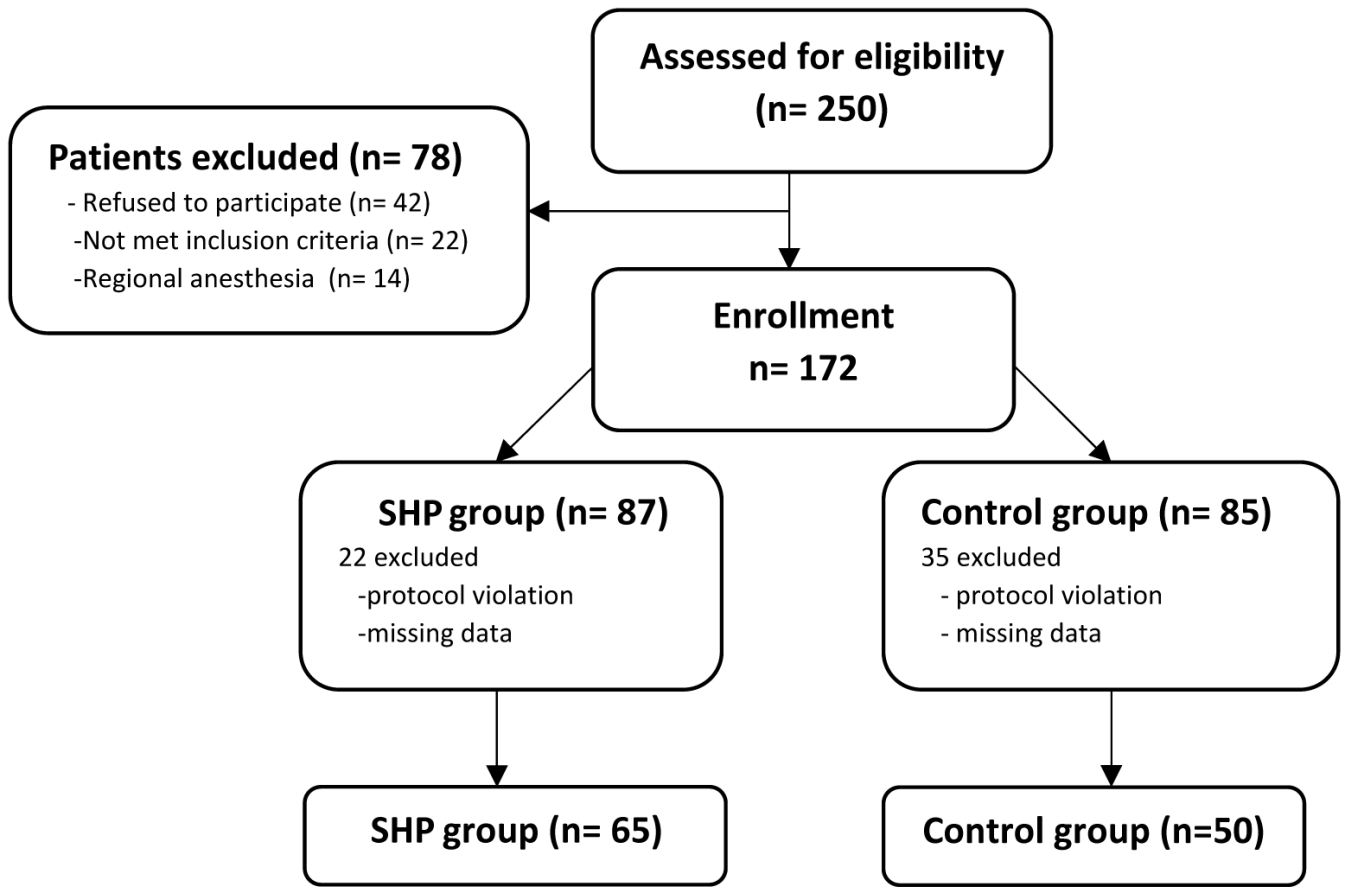
Flowchart of the study. SHPB – superior hypogastric plexus block.

The study group consisted of 65 patients and the control group consisted of 50 patients, a total of 115 patients (Figure 2). The mean age was 30.7 ± 6.0 (min-max: 18-46). Patient characteristics are shown in Table 1. The groups did not significantly differ in age, body height, body weight, BMI, and gravidity.

**Table 1:** The characteristics of patients who received superior hypogastric plexus (SHP) block for postoperative pain relief after cesarean section and controls, as well as primary and secondary outcomes of the study

	Control group (n = 50)			SHP block group (n = 65)			P	z/X^2^
	Mean ± standard deviation	Median	1st Q-3rd Q	Mean ± standard deviation	Median	1st Q-3rdQ		
**Age (years)**	30.6 ± 6.0	31.5	25.0-37.0	30.9 ± 6.0	31.0	26.0-36.0	0.881	-0.150*
**Body weight (kg)**	78.6 ± 11.7	80.0	70.0-85.3	77.4 ± 11.8	78.0	70.0-85.0	0.525	-0.636*
**Body height (cm)**	161.4 ± 5.1	160.0	157.8-165.0	162.7 ± 6.2	162.0	158.5-167	0.257	-1.134*
**BMI (kg/m^2^)**	30.2 ± 4.5	29.9	27.5-32.2	29.3 ± 4.2	29.4	26.3-32.4	0.404	-0.835*
**Gravidity (n)**	3.2 ± 1.9	3.0	2.0-4.0	2.6 ± 1.3	2.0	2.0-3.0	0.087	-1.713*
**Duration of operation (min)**	44.3 ± 7.7	42.0	39.8-50.0	50.3 ± 6.8	50.0	45.0-55.0	<0.001	-4.040*
**Postoperative Visual Analog Scale score**								
**at rest**								
**2 hours**	8.1 ± 1.6	8.0	8.0-9.3	5.4 ± 1.8	6.0	6.0-6.0	<0.001	-7.376*
**6 hours**	6.3 ± 2.0	6.0	5.8-8	3.9 ± 1.4	4.0	4.0-4.0	<0.001	-6.057*
**24 hours**	3.5 ± 2.0	4.0	2.0-4.3	2.3 ± 1.6	2.0	2.0-4.0	0.002	-3.064*
**48 hours**	5.0 ± 1.4	4.0	4.0-6	2.0 ± 0.6	2.0	2.0-2.0	<0.001	-9.255*
**on movement**								
**2 hours**	8.7 ± 1.2	9.0	8.0-10.0	6.5 ± 1.3	6.0	6.0-8.0	<0.001	-7.362*
**6 hours**	7.4 ± 1.7	8.0	6.0-8.0	5.6 ± 2.1	6.0	4.0-7.0	<0.001	-4.376*
**24 hours**	4.7 ± 1.8	5.5	4.0-6.0	3.2 ± 1.7	2.0	2.0-4.0	<0.001	-4.297*
**48 hours**	3.9 ± 1.5	4.0	2.0-4.0	2.0 ± 1.2	2.0	2.0-2.0	<0.001	-6.483*
**First passing of gas (hours)**	18.6 ± 9.1	16.0	12.0-24.0	14.7 ± 6.4	14.0	12.0-18.0	0.081	-1.745*
**NSAID requirement (n of vials)**	1.8 ± 1.3	1.0		0.9 ± 0.9	1.0		<0.001	-4.090*
**NSAID requirement, n (%)**	n = 50 (100)			n = 65 (100)				
**no**	3 (6.0)			22 (33.9)			<0.001	6.678
**yes**	47 (94.0)			43 (66.1)				
**Opioid required; n (%)**								
**No opioid use**	26 (52.0)			56 (86.2)			<0.001	16.221
**1 vial**	20 (40.0)			7 (10.8)				
**2 vials**	4 (8.0)			2 (3.1)				
**Nausea, n (%)**								
**no**	32 (64.0)			58 (89.2)			0.001	10.574
**yes**	18 (36.0)			7 (10.8)				
**Vomiting, n (%)**								
**no**	41 (82.0)			62 (95.4)			0.020	5.417
**yes**	9 (18.0)			3 (4.6)				

*Mann-Whitney U test.

### VAS score outcomes

**Figure 3:**
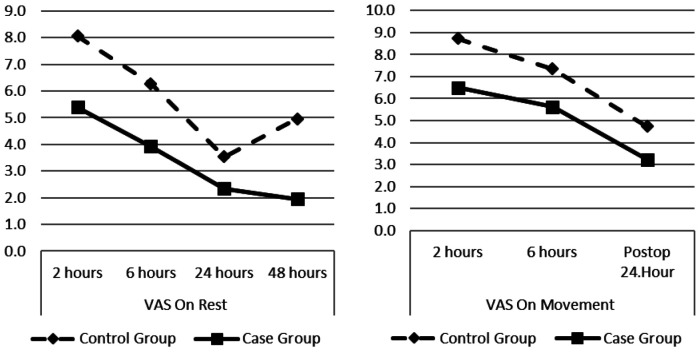
Visual analog scale scores on movement and at rest in patients who received superior hypogastric plexus (SHP) block for postoperative pain relief after a cesarean section and in controls.

**Figure 4:**
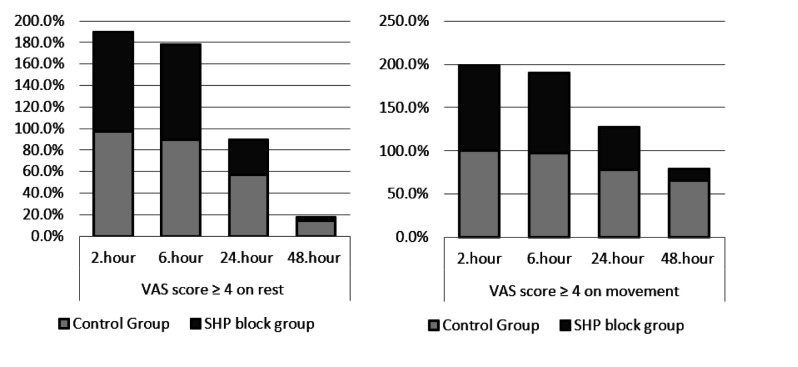
Visual analog scale scores ≥4 on movement and at rest in patients who received superior hypogastric plexus (SHP) block for postoperative pain relief after a cesarean section and in controls.

The study group had significantly lower VAS scores at rest and on movement 24 hours after surgery (*P* = 0.002, *P* < 0.001 respectively), as well as 2, 6, and 48 hours after surgery (*P* < 0.001) (Figure 3, Table 1). The number of patients with VAS<4 on movement and at rest 24 and 48 hours after surgery was significantly higher in the SHP block group (*P* < 0.05) (Figure 4).

### NSAID and opioid outcomes

**Figure 5:**
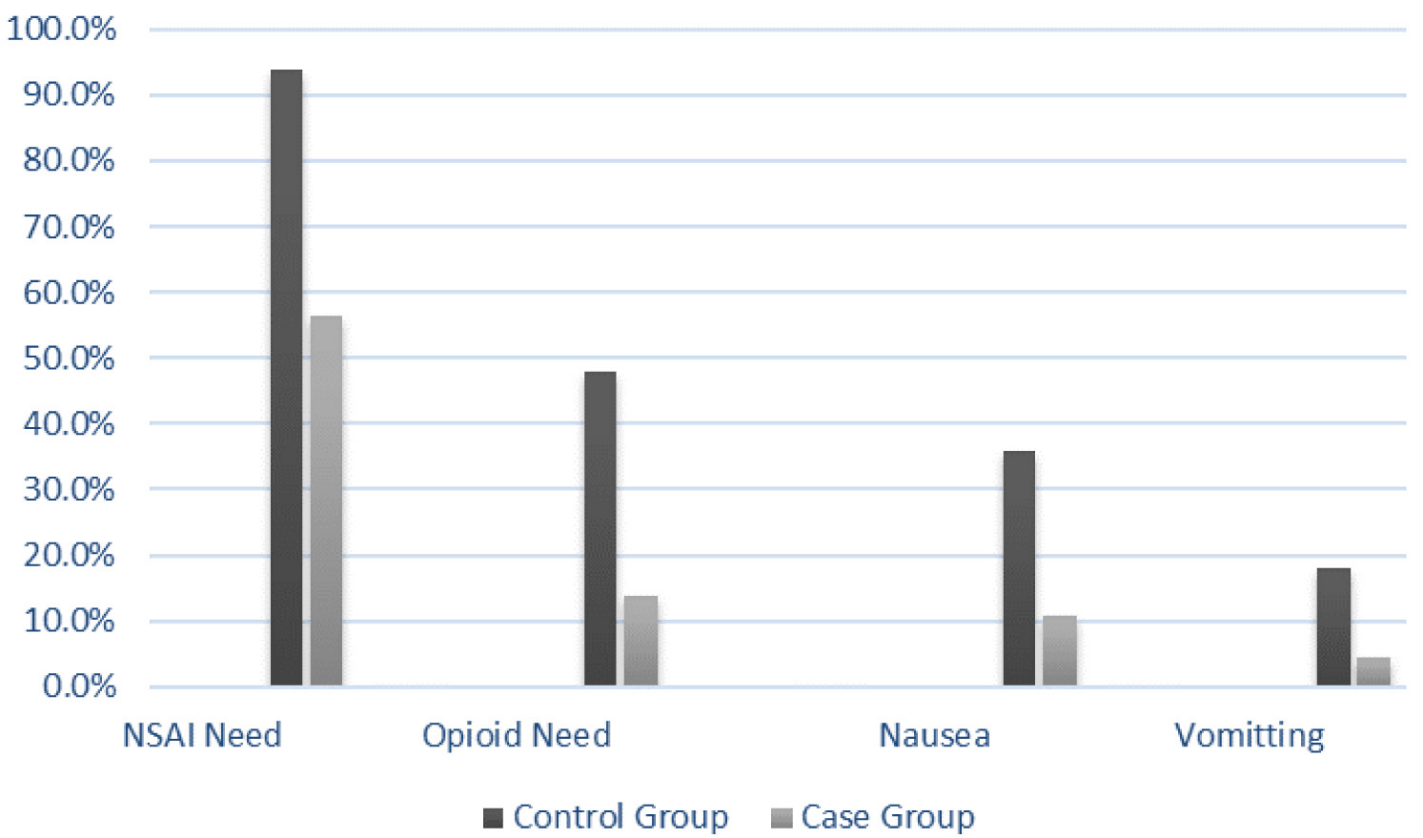
Nonsteroid antiinflammatory drug (NSAID) requirement, opioid requirement, nausea and vomiting in patients who received superior hypogastric plexus (SHP) block for postoperative pain relief after a cesarean section and in controls.

In the control group, 94% of the patients required rescue NSAID dose and 48% required opioids for analgesia. In the study group, these rates were only 56.5% and 13.9%, respectively. The study group needed a significantly lower rescue dose of NSAIDs or opioids (*P* = 0.003, *P* < 0.05, respectively). They also had a significantly lower incidence of nausea and vomiting (*P* < 0.05) (Figure 5), significantly longer surgery (*P* < 0.001), and significantly shorter time to the first passing of gas after surgery (*P* = 0.009).

## DISCUSSION

In this study, SHP block was shown to effectively control pain after a cesarean section. Postoperative pain scores and rescue dose analgesic requirement significantly decreased in the study group, while no complications and side effects were reported.

The main purpose of postoperative pain relief after cesarean section should be to safely and effectively provide maximum pain control with minimal side effects to the mother and baby. A few studies have investigated SHP block in patients undergoing a hysterectomy (11-13). Aytuluk et al reported that patients who were administered SHP block when undergoing laparoscopic hysterectomy had significantly lower VAS scores 1, 6, 12, 24, and 48 hours postoperatively (12). We also found significantly lower VAS scores in the SHP block group 2, 6, 24, and 48 hours postoperatively after a cesarean section. Rapp et al found that in patients with hysterectomy SHP block reduced VAS scores, especially 2 hours post-injection (11). However, after 6 hours the VAS scores did not significantly differ between the groups. This finding was expected since all patients received extra pain relief. Similarly, another study reported significantly lower VAS scores in patients undergoing abdominal hysterectomy with SHP block (13). A recent study found SHP block to effectively control postoperative pain in women who underwent a cesarean section, which is consistent with the present results (17).

Postoperative pain has a visceral and a somatic component. SHP block is mainly used to control the visceral pain (18). Accordingly, adding other nerve block techniques for somatic pain control (ie, transversus abdominis plane block, ilioinguinal/iliohypogastric block) or combining them with abdominal wall blocks may provide more effective pain relief. In this regard, Carney et al (19) suggested transversus abdominis plane block as a method for postoperative pain relief in patients undergoing hysterectomy.

SHP block was first introduced in 1990 in patients with pelvic pain secondary to cancer (20). The complications include neuraxial injection, discitis, interosseous or intravascular injection, or intra-abdominal organ injury. Since the SHP contains nerve fibers extending to the bladder, urinary incontinence or neurogenic bladder may also occur (14). None of these complications were observed in our patients. No motor block and no side effect related to bupivacaine-like sympathetic blockage and hemodynamic instability (hypotension, bradycardia) were recorded. Other studies also did not report any complications (11-13). According to our results, SHP block added only seven minutes on average to the length of surgery. SHP block was shown to be an easily applicable and safe method to perform intraoperatively.

Some authors reported no difference in the rates of postoperative nausea and vomiting between the SHP block group and controls (11,12). In contrast, we found significantly lower postoperative nausea and vomiting rates in the SHP block group. Similarly, Mahmood et al (13) observed a lower rate of vomiting in the SHP block group. In our study, bowel function returned significantly earlier in the SHP than in the control group, contrary to the results by Rapp et al (11). Earlier bowel function return might be explained by more comfortable movement of patients who were in less pain. We found the SHP block group to have a significantly more patients with a VAS score lower than four 24 and 48 hours after surgery. Similarly, Rapp et al (11) reported a significantly higher rate of women with a VAS score <4 two hours after injection in the SHP block group compared with the placebo group. Consistent with the literature, in our study the SHP group had significantly lower analgesic requirements than controls (11-13). Opioids are still the cornerstone of treatment of moderate to severe postoperative pain despite concerns about their use (21). They have many adverse effects, such as nausea, vomiting, constipation, respiratory depression, and may even be life-threatening (22). They may also negatively affect the health of the newborns of mothers who receive this type of treatment. Previous studies showed lower neonatal neurobehavioral scores when opiates were administered postpartum, as opiate metabolites accumulate in the colostrum (23). In the present study, SHP block had a significant morphine-sparing effect. The SHP group had lower opioid consumption, which resulted in a lower incidence of opioid-related adverse effects for the mother and newborn.

Pain control allows mothers to breastfeed more comfortably and care better for the baby (10). Severe postoperative pain after a cesarean section is a risk factor for postpartum depression (5). Moreover, the recovery quality closely correlates with pain relief (24). In this study, the SHP group had better pain scores and fewer complications, and needed less analgesic medications for pain control.

This is one of the rare studies investigating the efficacy of the SHP block after a cesarean section. The main study strength is the inclusion only of patients undergoing primary elective cesarean section. This enabled us to more homogeneously evaluate pain scores. As a limitation, we performed the SHP block only in patients under general anesthesia to clearly observe the efficacy of the method. However, considering the wide use of spinal anesthesia in cesarean sections, further studies with both types of anesthesia are needed. This intervention should also be assessed in various populations, such as in women with previous surgery. We also did not collect the data on long-term complications of the procedure.

In conclusion, the SHP block may be a safe alternative analgesic method for use after a cesarean section. This method not only lowered the postoperative pain scores, but also showed fewer side effects compared with opioids. Still, larger studies are needed to confirm these results.
